# Early ependymal tumor with MN1-BEND2 fusion: a mostly cerebral tumor of female children with a good prognosis that is distinct from classical astroblastoma

**DOI:** 10.1007/s11060-022-04222-1

**Published:** 2023-01-06

**Authors:** Norman L. Lehman

**Affiliations:** Departments of Pathology and Laboratory Medicine, Biochemistry and Molecular Genetics, and the Brown Cancer Center, University of Louisville, 505 S Hancock St, Louisville, KY 40202 USA

**Keywords:** Early ependymal tumor with MN1-BEND2 fusion, HGNET BCOR ex15 ITD, EWSR1-BEND2, NET-PATZ1, NET-MN1, MN1-CXXC5, Astroblastoma, Cerebral tumor, Supratentorial ependymoma, Pediatric, Female, Good prognosis, Genomic DNA methylation, Chromatin remodeling

## Abstract

**Purpose:**

Review of the clinicopathologic and genetic features of *early ependymal tumor with MN1-BEND2 fusion* (EET MN1-BEND2), classical astroblastomas, and recently described related pediatric CNS tumors. I also briefly review general mechanisms of gene expression silencing by DNA methylation and chromatin remodeling, and genomic DNA methylation profiling as a powerful new tool for CNS tumor classification.

**Methods:**

Literature review and illustration of tumor histopathologic features and prenatal gene expression timelines.

**Results:**

Astroblastoma, originally descried by Bailey and Cushing in 1926, has been an enigmatic tumor. Whether they are of ependymal or astrocytic derivation was argued for decades. Recent genetic evidence supports existence of both ependymal and astrocytic astroblastoma-like tumors. Studies have shown that tumors exhibiting astroblastoma-like histology can be classified into discrete entities based on their genomic DNA methylation profiles, gene expression, and in some cases, the presence of unique gene fusions. One such tumor, EET MN1-BEND2 occurs mostly in female children, and has an overall very good prognosis with surgical management. It contains a gene fusion comprised of portions of the *MN1* gene at chromosomal location 22q12.1 and the *BEND2* gene at Xp22.13. Other emerging pediatric CNS tumor entities demonstrating ependymal or astroblastoma-like histological features also harbor gene fusions involving chromosome X, 11q22 and 22q12 breakpoint regions.

**Conclusions:**

Genomic DNA profiling has facilitated discovery of several new CNS tumor entities, however, traditional methods, such as immunohistochemistry, DNA or RNA sequencing, and cytogenetic studies, including fluorescence in situ hybridization, remain necessary for their accurate biological classification and diagnosis.

**Supplementary Information:**

The online version contains supplementary material available at 10.1007/s11060-022-04222-1.

## Epigenetic regulation of gene expression and genomic DNA methylation analysis

Nuclear chromatin is comprised of histones and other proteins wrapped within coils of genomic DNA forming nucleosome structures that are either transcriptionally active (euchromatin) or inactive (heterochromatin). Differential gene expression occurs through multiple mechanisms but is largely due to epigenetic silencing of genes by DNA methylation [1]. Methylation of cytosine residues at multiple CpG dinucleotide sites within gene promoters, and adjacent first exons, effectively turns off gene expression by directly hindering binding of some transcription factors, and by recruiting proteins that alter chromatin structure and further interfere with transcription. Such proteins include methyl-CpG binding proteins that both repress transcription directly and recruit histone deacetylases (HDACs) and other transcriptional corepressors. Deacetylation of specific histone amino-terminal lysine residues restricts transcription factor access to DNA, while acetylation is permissive to transcription [2]. Histone lysine methylation^1^ by histone methyltransferases (HMTs), e.g., histone 3 (3.1, 3.2 and 3.3) lysine 28^2^ trimethylation (H3K28me3), represses gene transcription by promoting heterochromatin assembly [4], or can activate transcription when less histone methylation, e.g., histone 3 lysine 28 monomethylation (H3K28me1), and/or concomitant acetylation occurs [5–7]. Missense mutation of H3K28 to methionine (H3K28M, aka H3K27M) in diffuse midline glioma leads to decreased histone methylation at this site [8].

**Fig. 1:**
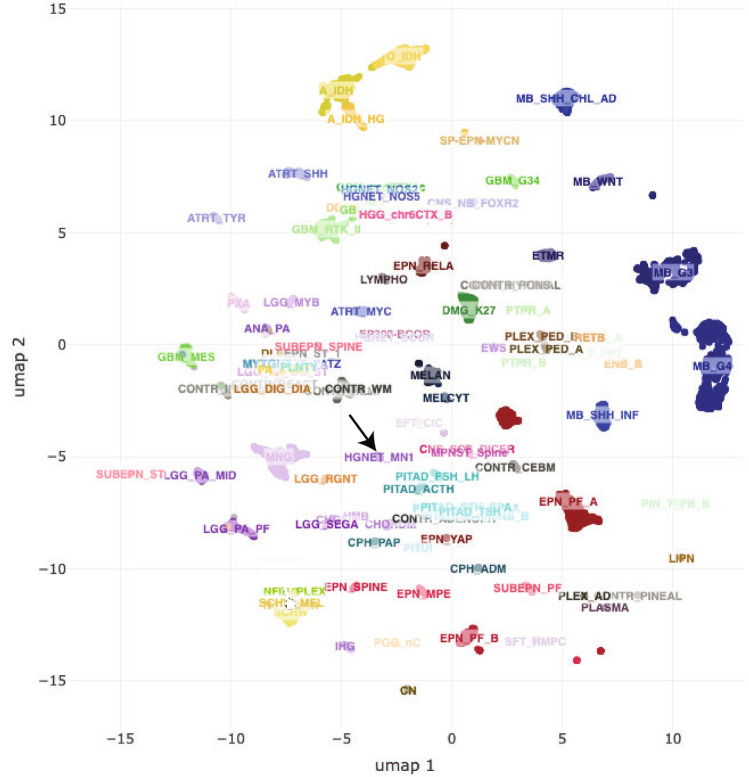
Unsupervised UMAP dimension reduction analysis map of genomic DNA methylation from the DFKZ CNS tumor reference set. The reference set includes methylation data for 2729 tumors (76 pathological diagnoses) and 72 normal brain tissues. Each group of data points represent a specific normal tissue or tumor diagnosis or closely related diagnoses [9]. The arrow indicates HGNET MN1 tumors (consisting mostly of EET MN1-BEND2)

Several new central nervous system (CNS) tumor entities have been defined based on genomic DNA methylation profiling using Illumina’s Methylation450K and MethylationEPIC BeadChips [9–11]. This technology detects methylated cytosine residues (5-methyl-cytosine) in CpG sites and can be used to profile genomic DNA methylation from archived formalin-fixed, paraffin-embedded (FFPE) resected tumor material [9, 10, 12, 13]. DNA is extracted from an FFPE tissue block and treated with bisulfite to convert unmethylated cytosines to uracil. Amplification of the DNA replaces uracil with thymidine. Following amplification and fragmentation, the DNA is hybridized to the MethylationEPIC BeadChip, which contains methylation status-specific oligonucleotide probes for over 850,000 methylation sites. Further processing steps and data analysis allow comparison of an individual tumor’s genomic DNA methylation profile to those of known tumors in a reference set using a random forest brain tumor classifier developed by the German Cancer Research Center (DKFZ, www.molecularneuropathology.org/mnp/) [9] and/or by data dimensionality reduction algorithms, e.g., t-distributed Stochastic Neighbor Embedding (tSNE) or Uniform Manifold Approximation and Projection (UMAP) followed by two- or three-dimensional mapping [9–14] (Fig. 1).

## Early ependymal tumor with *MN1-BEND2* fusion

**Fig. 2:**
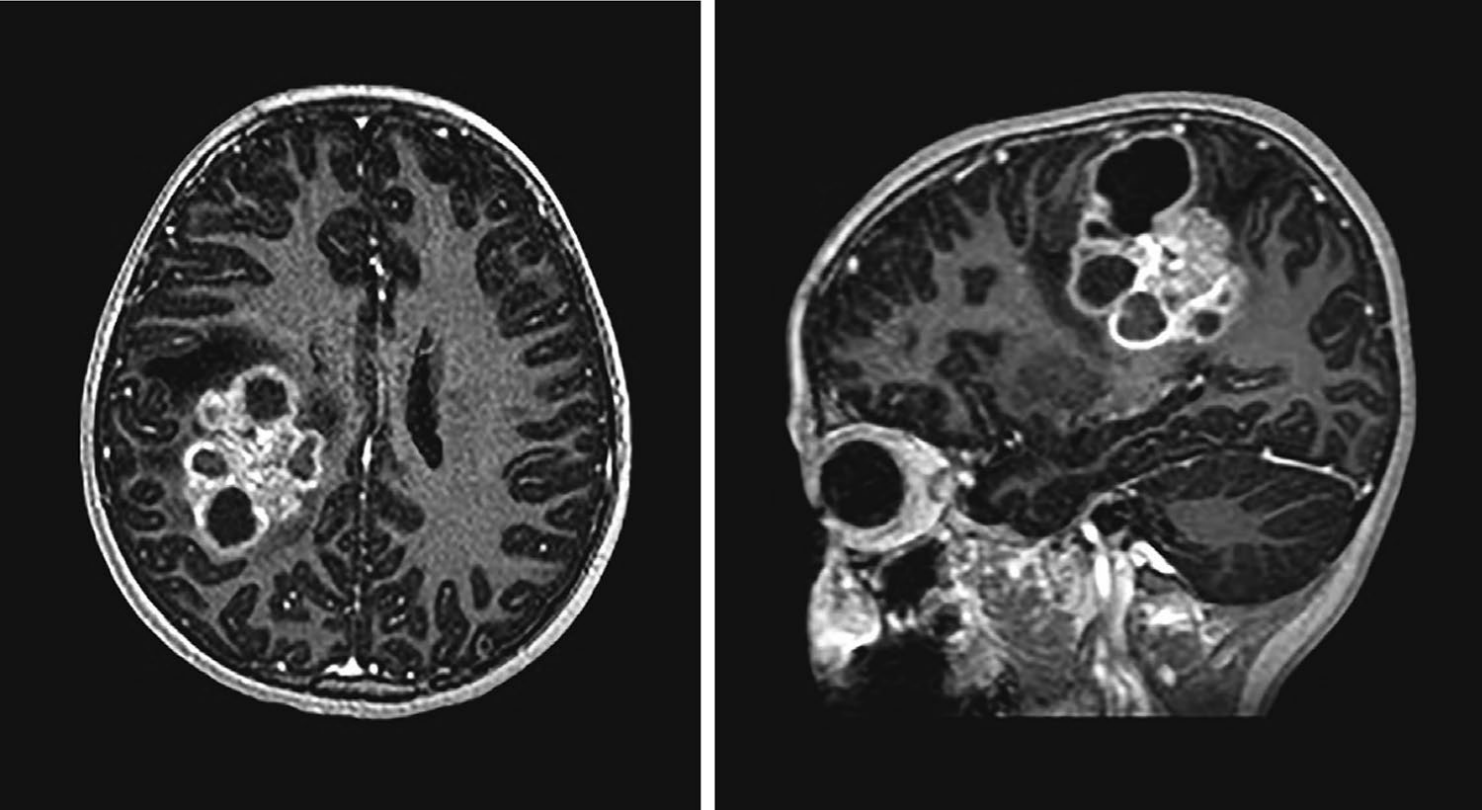
Typical MR findings of EET MN1-BEND2. T1 post-contrast MR images of EET MN1-BEND2 presenting in a 9-year-old girl. T1 post-contrast axial and sagital images show typical well-demarcated complex solid and cystic appearance of EET MN1-BEND2. Like other supratentorial ependymal tumors, they often show a bubbly and/or multinodular appearance [59]. Images courtesy of Dr. Bret Mobley, Vanderbilt University

*Early ependymal tumors with MN1-BEND2 fusion* (EET MN1-BEND2) are pediatric cerebral tumors most often occurring in the parietal or frontal lobes [10], however, a spinal tumor containing the fusion has also been described (Fig. 2, Table 1, Table S1). To date, cases with confirmed *MN1-BEND2* fusions have only been well documented in females (n = 19), the vast majority in children (mean and median ages, 9.6 and 9 years, respectively, n = 18) [10] (Table S1). However, larger studies are needed to confirm their sex distribution.

**Table 1:**
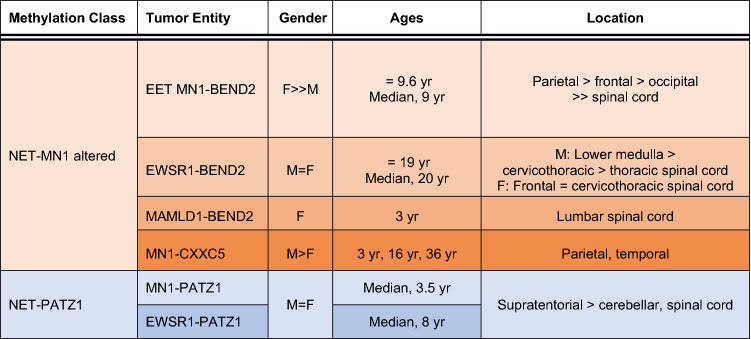
Methylation classes and clinical characteristics of ETT MN1-BEND2 and related tumors

Different shades of the same color indicate methylation subclasses

*yr* years, $\overline{x }$ mean

EET MN1-BEND2 was first identified as a subset of pediatric CNS tumors belonging to a methylation class defined as *high-grade neuroepithelial tumors with MN1 alteration* (HGNET-MN1). This designation was based on DNA methylation profiling combined with identification of *MN1-BEND2* and *MN1-CXXC5* fusions by RNA-seq in a small number of cases, and detection of nonspecific *MN1* gene rearrangement by break-apart fluorescence in situ hybridization (FISH) [13].

The protein encoded by the *meningioma (disrupted in balanced translocation) 1* (*MN1*) gene at chromosome 22q12.1 acts as a chromatin remodeler and transcriptional coregulator [15]. The function of *BEN domain containing 2* encoded by *BEND2* at Xp22.13 is unknown. However, other BEN domain-containing DNA-binding proteins are involved in chromatin remodeling [16].

Studies from our group confirmed the presence of *MN1-BEND2* fusions in additional tumors within the HGNET-MN1 *methylation class* and demonstrated that they are typically associated with patient survival of over 10 years [10, 12]. Clearly, many tumors within this methylation class are not clinically high-grade. Therefore, I will henceforth refer to it as *neuroepithelial tumors with MN1 alteration* (NET-MN1).

In our study, the 5-year (n = 6)^3^ and 10-year (n = 5) survival values for EET MN1-BEND2 were both 100% [12]. Tumor recurrences requiring re-resection were relatively common, however. One-half of patients experienced recurrence after initial resection: one patient at 4.3 years; one at 2, 4 and 4.5 years; and one at 1, 4, 5, 8 and 11 years.

**Fig. 3:**
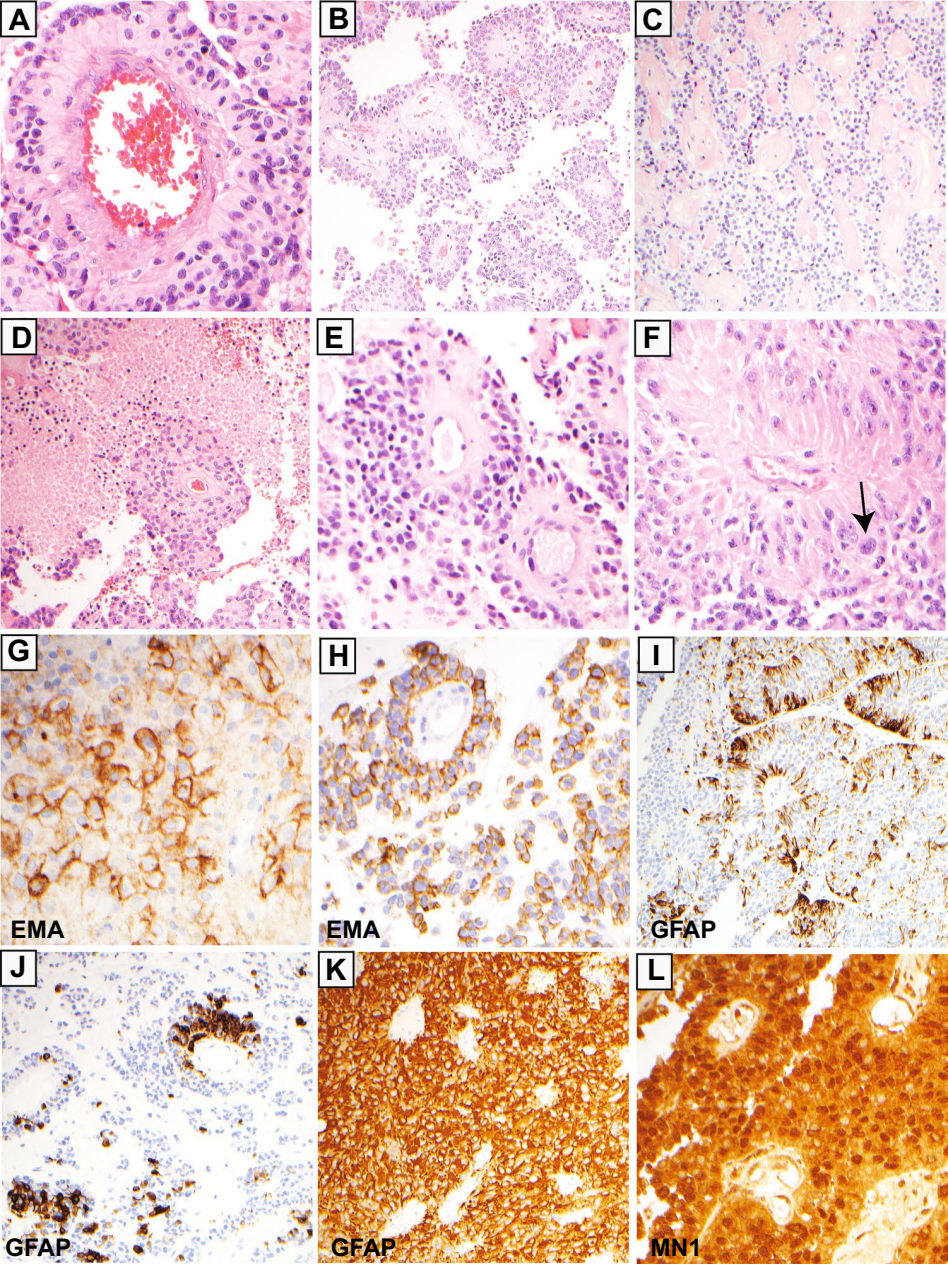
Histopathology of EET MN1-BEND2. **A** Perivascular pseudorosette in EET MN1-BEND2**, B** EET MN1-BEND2 papillary growth pattern. **C** Vascular and stromal sclerosis in EET MN1-BEND2. **D** EET MN1-BEND2 showing tumor necrosis. **E** ZFTA-RELA supratentorial ependymoma pseudorosette. **F** MAPK astroblastoma pseudorosette demonstrating more elongated cells with prominent nucleoli. A multinucleate cell is indicated by the arrow. **G** EET MN1-BEND2 EMA immunohistochemical stain demonstrating membrane and dot-like cytoplasmic positivity. **H** ZFTA-RELA supratentorial ependymoma showing similar EMA immunostaining. **I **and** J** Scattered GFAP immunostaining in EET MN1-BEND2 and ZFTA-RELA ependymoma, respectively. **K** Diffuse GFAP staining in MAPK astroblastoma. **L** MN1 immunohistochemical stain of EET MN1-BEND2 depicting strong nuclear staining. Such staining was absent in MAPK astroblastomas, however the sensitivity and specificity of MN1 immunohistochemical staining for EET MN1-BEND2 is not yet known [10]

Histologically, EET MN1-BEND2 are characterized by abundant perivascular tumor cell pseudorosettes arranged in a solid and/or loose papillary pattern corresponding to solid and cystic components by imaging, respectively [12, 17] (Figs. 2, 3B and C). EET MN1-BEND2 are generally well circumscribed both radiographically and microscopically and generally do not infiltrate brain parenchyma. Tumor blood vessels are often hyalinized and can appear sclerotic, as may the intervening tumor stroma (Fig. 3C). Mitotic activity and necrosis are frequently present (Fig. 3D). In addition to polygonal, columnar, and sometimes tapered perivascular tumor cells, focal clear or rhabdoid cytomorphology may also occur [10, 17–20]. Immunohistochemistry reveals that EET MN1-BEND2 usually demonstrate cell membrane and dot-like cytoplasmic *epithelial membrane antigen* (EMA, aka MUC1) and *podoplanin* immunoreactivity, and variable patchy or negative *glial fibrillary acidic protein* (GFAP) immunoreactivity [10, 19, 21, 22] (Fig. 3G and I). These histologic and immunohistochemical features are also found in other supratentorial and spinal ependymal tumors [18, 23–27] (Fig. 3E, H and J).

EET MN1-BEND2 were termed *astroblastoma, MN1 altered* in the 2021 WHO classification of CNS tumors [18] because of their general resemblance to astroblastomas originally described by Percival Bailey, Harvey Cushing, and Paul Bucy [28, 29], that is, their demonstration of numerous, often back-to-back tumor cell perivascular pseudorosettes, sometimes referred to as *astroblastic pseudorosettes*. The latter are similar to, and at times indistinguishable from, pseudorosettes seen in supratentorial or spinal ependymomas [13, 17, 23, 30] (Fig. 3A, B and E).

**Fig. 4:**
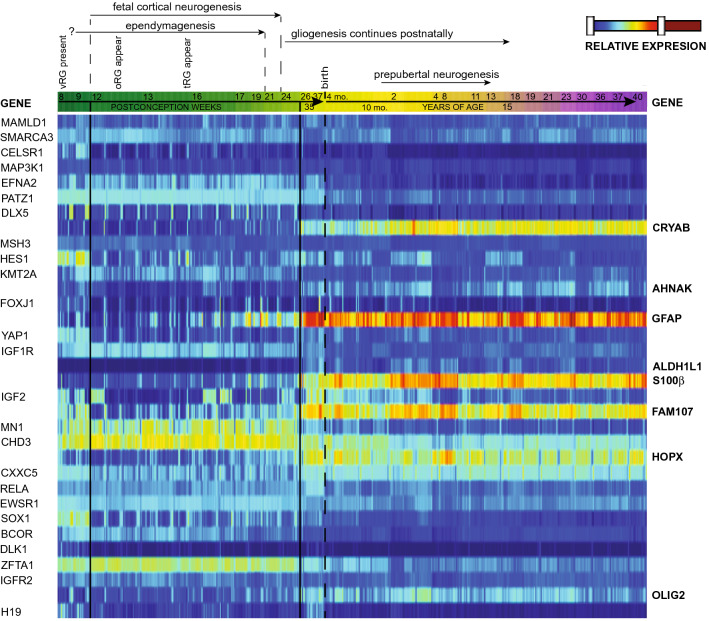
Pediatric supratentorial ependymal tumor genes are highly expressed in the late embryonic/early fetal period and classical astroblastoma associated genes are expressed later during fetal and postnatal gliogenesis. The developmental expression timecourse (in post-conception weeks) of select genes overexpressed or mutated in EET MN1-BEND2 and other pediatric supratentorial ependymomas and MAPK astroblastomas was obtained from the Allen Human Developmental Transcriptome database. Pediatric supratentorial ependymoma and related tumor genes (relatively overexpressed or mutated) are depicted in plain type on the left and MAPK astrocytoma associated genes are in bold on the right. The patient age bar progressive color scheme is arbitrary. Pediatric ependymal tumor associated genes, including *MAMLD1*, *PATZ1*, *FOXJ1*, *YAP1*, *MN1*, *CXXC5*, *RELA*, *EWSR1*, *BCOR* and *ZFTA1* are more highly expressed prior to 25 pcw. Some EET MN1-BEND2 associated genes, e.g., *CELSR1*, *DLX5*, *HES1*, *FOXJ1*, *YAP1*, *SOX1*, *BCOR* and *H19*, are most highly expressed prior to 10 pcw during the late embryonic/early fetal period. MAPK astrocytoma associated genes are more highly expressed after 25 pcw. Transcript expression is normalized by reads per kilobase of transcript per million mapped reads (RPKM) to compensate for RNA-seq generation of more sequencing reads from longer RNA molecules. Data is from up to 16 brain regions from 42 specimens [Allen Institute for Brain Science. Allen Human Brain Atlas. BrainSpan: Atlas of the Developing Human Brain—Developmental Transcriptome, 2010; https://www.brainspan.org/rnaseq/search/index.html (Accessed 10/31/2022)]

Colleagues and I have recently shown that the gene expression profile of EET MN1-BEND2 strongly suggests ependymal differentiation, particularly derivation from an early ependymal precursor, and not astrocytic differentiation or derivation from an astrocyte precursor as “astroblastoma” implies [10]. EET MN1-BEND2 express high mRNA levels of the ependymoma-associated genes *FOXJ1*, *IGF2*, *CELSR1*, *RFX3, KCNJ5*, *TFF3* and *YAP1* and relatively low levels of messages of canonical astrocyte marker genes such as *OLIG2, GFAP, ALDH1L1*, and *S100 β* [10, 31]. EET MN1-BEND2 are also enriched for homeobox gene expression, e.g., *CUX2*, *SHOX*, *SOX1, SOX14*, *IRX2*, *PAX1*, *HOXD10*, *DLX5* and *PRRX2*, and for *HES1*, *H19*, and the ATP binding cassette transporter gene *ABCC1* encoding *multidrug resistance-associated protein 1*. These genes are highly expressed during embryonic and fetal development by the primitive neuroepithelium and/or ventricular zone radial glia (vRG) neural stem cells, the latter of which conventional ependymomas are believed to be derived from [10, 32, 33]. We have thus defined these tumors as early ependymal tumors with MN1-BEND2 fusion due to their expression of both early neural stem/progenitor cell and canonical ependymoma genes (Fig. 4).

## Additional genetic features of EET MN1-BEND2

In addition to *MN1* and *BEND2*, overexpression or mutation of additional genes involved in chromatin remodeling were found in EET MN1-BEND2 tumors, e.g., in the SWI/SNF complex genes *SMARCA1*, *SMARCAD1* and *alpha-thalassemia, X-linked mental retardation* gene (*ATRX*), and in *lysine methyltransferase 2A* (*KMT2A*, aka *MLL1*) [10]. The latter is a histone methyltransferase and transcriptional coactivator important in chromatin structural regulation and neural progenitor proliferation [6]. It is a subunit of the MLL1/MLL multiprotein complex that mediates both methylation of histone 3 lysine 4 (H3K4me) and acetylation of histone 4 lysine 16 (H4K16ac) [34]. EET MN1-BEND2 additionally overexpressed *CHD3* encoding a component of the Mi-2/NuRD histone deacetylase complex. The mismatch repair gene *MSH3* (at chromosome 5q14.1) was also frequently mutated in EET MN1-BEND2 [10].

Chromosomal copy number variations (CNVs) found in EET MN1-BEND2 include loss of portions of chromosomes 6, 8, 9, 10, 14, 16, 18, 22q and X, and gains of 6p, 9p and X, however, the most frequent CNVs observed were losses of chromosomes 14, 16, 22q and X [12]. Notably, 22q loss is the most common chromosomal abnormality in ependymomas [35].

Additional genes highly expressed in EET MN1-BEND2 include the ventricular zone radial glia-enriched gene *H19*, which is implicated as a tumor suppressor in the pediatric neoplasm Wilms tumor. The *H19* gene product is a long noncoding RNA required for the recruitment of *methyl-CpG-binding domain protein 1* (MBD1) and thus histone deacetylase to methylated sites on the nearby *insulin growth factor 2* (*IGF2*) gene, resulting in its decreased transcription. miR483, also overexpressed in Wilms tumor and EET MN1-BEND2, enhances transcription of *IGF2* and *IGF2 antisense* (*IGF2-AS*) genes. *FAM3B*, whose protein is involved in insulin secretion and apoptosis of insulin secreting cells, was also overexpressed. Like others, we found that *MUM1*, which facilitates DNA damage repair-associated chromatin changes, appears highly overexpressed in EET MN1-BEND2, however this was not the case when compared to normal brain controls [10, 36].

## MAPK pathway activated classical astroblastomas

Other tumors also traditionally called astroblastomas more highly express *OLIG2, GFAP, ALDH1L1*, and *S100 β* astrocyte genes and exhibit histomorphologic and patient demographic characteristics more closely matching original descriptions of astroblastoma [10, 28]. These astrocyte-like astroblastomas are associated with intermediate-grade biological behavior and occur in male children, and young to middle-aged adults (rarely older adults) of both sexes [10, 12]. They demonstrate genomic methylation patterns similar, but not identical, to those of pleomorphic xanthoastrocytoma (PXA). Like PXA and other astrocytomas, they highly express and frequently harbor mutations in *mitogen activated protein kinase* (MAPK) pathway genes, e.g., *BRAF*, *MAP3K5, MAP4K4* and *NF1*. *BRAF V600E* mutation especially occurred in young adult female patients [10, 12, 37].

*MAP3K1*, however, exhibited deletion mutations more often in EET MN1-BEND2 and other supratentorial ependymal tumors including *ZFTA-RELA* ependymomas and papillary tumors of the pineal region (PTPR). The latter share other genetic features with ependymomas and are considered to be ependymal tumors by some authors [10, 24, 38]. *MAP3K1* is a cell survival/apoptosis regulator involved in ERK and JNK MAPK pathways, and in NF-κB and p53 signaling. It was also mutant in an EET MN1-BEND2 case reported by others and its gene promoter is hypermethylated in pediatric supratentorial and spinal ependymomas [21, 39].

A large subset of MAPK astroblastomas also showed PI3K/AKT/mTOR pathway alterations, including *phosphoinositide-3-kinase* (*PI3K)* subunit (*PI3KCA*, *PI3KC3*, *PI3KR1*, and *PI3KR3*) overexpression or mutations, *AKT2, TSC2, RABEP1* and *PTEN* mutations, and *TERT* fusions not found in EET MN1-BEND2 [10]. They also harbor mutations in *AHNAK* (an actin binding protein related to phospholipase C signaling and cell migration), and more frequent mutations in WNT pathway genes, and show *TCF4* and *FAM107A* overexpression [10]. MAPK astroblastomas also demonstrate mutations in multiple chromatin regulatory genes, e.g., *SMARCA1*, *SMARCA2*, *SMARCAD1*, *SMARCD3*, *ATRX*, and in histone methyltransferases, e.g., *KMT2A*, *KMTA2C*, *KMT2D*, and *KMT2E*. Additionally, MAPK pathway-associated astroblastomas show greater gene expression overlap with cerebral astrocyte precursor cells, i.e., outer radial glia (oRG) and truncated radial glia (tRG), than do EET MN1-BEND2 [10, 40].

Histologically, MAPK astroblastomas more often demonstrate elongate, tapering perivascular cells and more prominent nucleoli than do EET MN1-BEND2 (Fig. 3F). These types of rosettes would more appropriately be described as “astroblastic” and morphologically resemble tRG. They also more frequently show multinucleate tumor cells, may exhibit eosinophilic granular bodies [10, 12, 17] and demonstrate invasion of neighboring brain parenchyma [17]. These histomorphologic features were also noted in the original descriptions of astroblastomas by Bailey and Cushing, and Bailey and Bucy [28, 29]. Unlike generally seen in ependymal tumors, MAPK astroblastomas are diffusely GFAP positive and EMA negative by immunohistochemical staining consistent with astrocyte differentiation (Fig. 3K). We suggest that the term astroblastoma should be reserved for these MAPK pathway dominant tumors (astroblastoma, MAPK type) [10].

## Other NET-MN1 methylation class tumors

The NET-MN1 methylation class contains two additional, closely associated subclasses of tumors characterized by *MN1-CXXC5* or *EWSR1-BEND2* fusions [13, 41, 42] (Table 1). *EWSR1* encodes *EWS RNA binding protein 1*, a transcriptional activator whose gene at 22q12.2 is also the first component of fusions found in the pediatric tumor Ewing sarcoma. *EWSR1-BEND2* harboring tumors tend to present in children to young adults [41] (Table 1, Table S1). Radiologic findings are similar to EET MN1-BEND2 [42]. Four cases presenting in the lower medulla and/or upper spinal cord have been reported in males: an infant aged 3 months (medulla to C4), a 20-year-old (brainstem NOS), a 38-year-old (lower medulla) and a 36-year-old (T3–T5) [41–44]. One cervicospinal and two frontal tumors with *EWSR1-BEND2* have been reported in females, aged 6, and 6 and 26 years, respectively [41]. The mean and median ages of reported cases with documented fusions are 19 and 20 years (n = 7), respectively. The *EWSR1-BEND2* fusion was also reported in a tumor described only as a spinal ependymoma [45]. Additionally, a cervicothoracic tumor with *EWSR1* rearrangement by FISH was reported in a 6-year-old girl [46] and a multiply recurrent cervicomedullary tumor matching the NET-MN1 methylation class, but not otherwise molecularly characterized, was reported in a woman who presented at approximately 16 years of age [47]. A pontomedullary tumor exhibiting vascular and stromal sclerosis and *MN1* rearrangement by FISH was reported in an 11-year-old male, possibly representing an EET MN1-BEND2 or *MN1-CXXC5* lesion [48]. 

The histologic features of *EWSR1-BEND2* tumors may be identical to those of EET MN1-BEND2*,* i.e., abundant perivascular pseudorosettes, including perivascular and stromal sclerosis in some cases, conspicuous mitotic activity, focal necrosis, patchy variable GFAP immunoreactivity, and often diffuse EMA positivity [41]. Overall survival of patients with *EWSR1-BEND2* tumors is less favorable than for EET MN1-BEND2, at approximately 60% at 5 years and likely attributable to their frequent medullary and upper spinal cord locations.

A lumbospinal tumor with *MAMLD1-BEND2* fusion matching the NET-MN1 methylation class, was reported in a 3-year-old girl [49]. *MAMLD1*, located at Xq28, encodes a developmentally important transcriptional coactivator [50]. *YAP1* at 11q22.1, encoding for a DNA-binding Hippo pathway regulatory protein, and *MAMLD1* fusions (*YAP1-MAMLD1*) appear to drive oncogenesis in a subset of supratentorial ependymomas occurring mostly in female infants [23, 51].

*CXXC5* at 5q31.2 codes for a protein that binds unmethylated CpG sites and promotes chromatin structural changes, thereby modulating expression of multiple proliferation, cell cycle arrest and cancer related genes [52]. Fewer *MN1-CXXC5* harboring tumors have been well described. One case, originally diagnosed as anaplastic ependymoma, presented in the temporal lobe of a 3-year-old boy [53], and another case in the parietal lobe of a 16-year-old male [13]. An additional case showing more poorly differentiated, tumor architecture lacking prominent pseudorosettes, occurred in the parietal lobe of a 36-year-old woman [54].

The extent in which *MN1-CXXC5*, *EWSR1-BEND2* and *MAMLD1-BEND2* containing tumors are biologically similar to EET MN1-BEND2 is not currently known. The histology of *MN1-CXXC5* tumors may vary compared to EET MN1-BEND2 and *EWSR1-BEND2* tumors, and indeed *MN1-CXXC5* tumors appear to form a slightly distant satellite cluster of the NET-MN1 methylation class [54]. The common denominator of *BEND2* as the downstream gene in *MN1-BEND2*, *EWSR1-BEND2* and *MAMLD1-BEND2* fusion harboring tumors has led to speculation that *BEND2* is the more biologically important overexpressed gene function in these histologically similar lesions [10, 41, 49]. Perhaps the NET-MN1 methylation class should be renamed NET-BEND2 altered.

## High-grade neuroepithelial tumor with BCOR exon 15 internal tandem duplication

High-grade neuroepithelial tumors with BCOR exon 15 internal tandem duplication *(HGNET BCOR ex15 ITD)* are rare pediatric tumors belonging to a discrete methylation class [13, 55]. BCOR (BCL6 co-repressor) at Xp11.4 represses gene transcription through interaction with the DNA binding protein BCL-6 [56] and may recruit a histone deacetylase. *BCOR* mutation, however, results in methylation of histone 3 lysines 4 and 36 leading to reactivated transcription of silenced genes [57].

HGNET BCOR ex15 ITD are predominantly cerebral tumors, but have also rarely occurred in the basal ganglia, cerebellum, and pons. From their series and literature review, Ferris et al*.* reported that they occur nearly equally in males and females (n = 35) in patients ranging from 0 to 22 years with a median patient age of 3.5 years [55]. Imaging shows large, well‐circumscribed, heterogeneous tumors demonstrating variable enhancement, often with central necrosis or hemorrhage, and restricted diffusion indicative of a highly cellular lesion [55].

The largest reported series of HGNET BCOR ex15 ITD describes them as being histologically heterogenous, but they typically contain more classical ependymoma-like pseudorosettes demonstrating a fibrillary perivascular anuclear zone, palisading necrosis, and an absence of microvascular proliferation [55, 58]. Homer Wright rosettes were additionally seen in some cases, which are not characteristic of ependymal tumors, but occur in more primitive embryonal tumors [58]. Although generally well circumscribed, some cases can be infiltrative. They are reportedly mostly GFAP-negative and lack ependymoma-like EMA immunoreactivity, however most cases demonstrate NeuN and BCOR nuclear immunoreactivity. EET MN1-BEND2 also highly express BCOR [10, 19]. HGNET BCOR ex15 ITD are truly high-grade tumors. Their prognosis appears to be significantly worse than that of EET MN1-BEND2, however, some long-term survivors are reported [55, 59]. Nosologically, they may be best considered an anaplastic early ependymal tumor or an embryonal tumor (Fig. 4).

## Neuroepithelial tumors with PATZ1 fusions

Neuroepithelial tumors with *PATZ1* fusions (NET-PATZ1) are a diverse group of mostly pediatric tumors harboring fusions between nearby chromosome 22q12 region genes, i.e., *MN1*-*PATZ1* or *EWSR1-PATZ1* [60] (Table 1). *PATZ1* at 22q12.2, like *MN1* at 22q12.1, encodes a chromatin remodeler and transcriptional coregulator. NET-PATZ1 tumors are relatively heterogenous histologically. Most were originally diagnosed as glioblastoma or high-grade astrocytoma, followed by anaplastic ependymoma. A cerebral tumor in a 13-year-old girl demonstrating a perivascular pseudorosette pattern harbored both *EWSR1-PATZ1* and *MN1-GTSE1* fusions [61]. *GTSE1* at 22q13.31 encodes a cell cycle regulatory protein that binds p53 and shuttles it out of the nucleus in response to DNA damage. In a separate report, a tumor with a *EWSR1-PATZ1* fusion was described as a ganglioglioma [62].

NET-PATZ1 frequently show necrosis, but like EET MN1-BEND2 and HGNET BCOR ex15 ITD, generally lack microvascular proliferation^4^. NET-PATZ1 appear to occur equally in male and female patients in multiple CNS locations (cerebrum, cerebellum, spinal cord), however most are supratentorial. Although, the demographic and anatomic data of NET-PATZ1 were not described individually for *MN1* and *EWSR1* fusion tumors [60]. tSNE analysis of their tumor methylation profiles reveals that both fusion types form a methylation class grouping, or perhaps separate, but closely associated subgroups [60]. Some NET-PATZ1, presumably those originally diagnosed as anaplastic ependymoma, show histology very similar to EET MN1-BEND2 [60]. NET-PATZ1 are predicted to show intermediate biological behavior. However, the latter is not established, and their clinical aggressiveness could be variable because of their overall heterogeneity.

## Diagnosis of EET BEND2 and related tumors

Although some features may be more common in one tumor type versus another within the extended NET-MN1 methylation class, HGNET BCOR ex15 ITD, other ependymal or astroblastoma-like tumors, and MAPK astroblastomas, these tumors cannot be reliably distinguished by histology alone [12]. Because it encompasses three or more distinct pathological entities, prior studies of cases assigned to the HGNET-MN1 methylation class should be interpreted with caution [59, 63–65].

Pathological diagnosis requires ancillary testing. Immunohistochemistry should be performed for EMA or podoplanin, GFAP, BCOR, and p65-RELA or L1CAM for *ZFTA-RELA* ependymomas [26, 66, 67]. Unlike MAPK astroblastoma and many astrocytomas, which tend to be diffusely GFAP immunoreactive, EET MN1-BEND2 shows variable, but usually only focal GFAP immunoreactivity, but like ependymoma, when present tends to be positive in perivascular pseudorosettes [10, 68, 69]. Like other ependymal tumors [70], NET-MN1 class lesions may occasionally show immunoreactivity for neuronal markers [59].

Currently, molecular studies are necessary to evaluate for characteristic fusions by FISH, PCR, RNA or DNA sequencing, or Nanostring technology [12, 21, 67, 71]. Genomic DNA methylation analysis will likely become increasingly helpful to establish a precise diagnosis of many pediatric CNS tumors [10, 11, 19]. FISH for *MN1-BEND2* using fusion probes rather than relatively nonspecific break apart analysis may be preferable [21]. More than one molecular diagnostic modality may be required, for example to confirm FISH or genomic DNA methylation results.

DNA methylation can be reliably performed on FFPE tissue, however, is currently only available at a limited number of academic clinical centers and is not yet FDA approved. Immunohistochemistry for MN1 is a promising cost-efficient procedure to help identify EET MN1-BEND2 tumors that can be easily performed in most medical centers (Fig. 3L). However, further studies are needed to establish its sensitivity and specificity, as it may be positive in other tumors highly expressing *MN1*, perhaps especially rare tumors with alternate *MN1* fusions.

## Treatment of EET MN1-BEND2 and related tumors

Treatment for EET MN1-BEND2 is primarily surgical. Complete resection should be pursued whenever possible, as it may be curative, offer the potential for very long-term patient survival, reduce morbidity and/or negate the need for adjuvant cytotoxic chemotherapy or potentially biotransformative radiation therapy [12, 18, 21].

Intraoperative pathological diagnosis or pre-resection stereotactic biopsy should be able to confirm ependymal histology and guide the surgical approach. A recent study demonstrated the feasibility of intraoperative tumor DNA methylation analysis, which could portend the future of intraoperative pathological diagnosis [72]. Fluorescence-guided resection using 5-aminolevulic acid (5-ALA) may be helpful to achieve gross total resection (GTR) [22].

As recurrence is common, long-term surveillance is necessary for EET MN1-BEND2 patients. Adjuvant therapy should be considered for patients with multiple recurrences and/or whose tumors are not completely resectable. For pediatric supratentorial ependymomas, GTR is associated with improved progression free survival, but not necessarily overall survival [69]. Conformational radiation (CRT) increases 5-year event free survival in ependymoma [73]. It would therefore be rational to treat EET-MN1 BEND2 and other new ependymal tumor entities with CRT if GTR is not possible. Complete resection may be hampered by the multinodular/multicystic nature of EET MN1-BEND2 and *EWSR1-BEND2* tumors. In brainstem or spinal cord tumors GTR may not be possible, therefore adjuvant therapy appears indicated [42, 74].

Confirmed and probable EET MN1-BEND2 cases have been treated with radiation or radiation and temozolomide with unclear benefits due to the variable, but overall indolent natural history of this entity [21, 22, 64]. Medullary and spinal cord related-tumors (e.g., *EWSR1-BEND2* lesions) that are not completely resectable have been successfully treated with radiation and temozolomide [42]. Yamada et al. [74] report a T1–T2 spinal cord astroblastoma-like tumor, with an apparent *MN1* tandem duplication by FISH, in a 20-year-old woman who exhibited dramatic functional improvement and tumor shrinkage in response to radiation, temozolomide, and bevacizumab. Because of EET MN1-BEND2’s overexpression of IGF2 pathway components and ABCC1, agents directed at these targets could be therapeutic candidates, perhaps in combination with radiation and temozolomide [10].

One EET MN1-BEND2 case presenting in a 6-year-old girl recurred multiple times over ten years and appeared to undergo malignant transformation with acquired mutations in NF-κB signaling proteins and increased expression of p65-RELA [21]. The patient was treated with radiation therapy and temozolomide after a second resection, and combined CCNU/temozolomide following a third. Therefore, transformation may have theoretically been treatment related.

## Summary

Astroblastoma has been a controversial entity. Some have argued they were of ependymal differentiation; others favored astrocytic derivation, while some have opined that astroblastoma histomorphology simply represents a nonspecific pattern [24]. We used the terminology early ependymal tumors with *MN1-BEND2* because of expression of early neural stem/progenitor cell and ependymoma-associated genes in this new tumor entity. They might also be appropriately called *ependymoma with MN1-BEND2*. Their histological features, immunohistochemical profile and generally noninvasive behavior overlap with established supratentorial ependymomas, as do those of related *EWSR1-BEND2* harboring tumors. These and other newly described pediatric astroblastoma- or ependymoma-like tumors should therefore probably be considered ependymal tumors.

Perhaps the most compelling reason for their classification as ependymal is that their current treatment and prognosis is more similar to that of other ependymal tumors than to that of astrocytic tumors. Neuroepithelial tumor is too broad a term as it can be used to describe any tumor ultimately derived from the primitive neuroepithelium, essentially all primary CNS tumors, and has thus become a “wastebasket” term [75]. Astroblastoma is similarly becoming a wastebasket description for several new tumor entities. Creation of additional tumor categories based on unique genetic features, e.g., specific gene fusions, that do not correlate with a truly novel histology or clinical behavior is not helpful, and medicine may be better served by considering such lesions subtypes of established lineages if they share similar overall genetics and biological behavior.

EET-MN1 patients may have very long-term survival despite the presence of intermediate to high-grade tumor histological features, i.e., mitotic activity, and necrosis, in many examples [12]. Similar to other supratentorial ependymal tumors, EET MN1-BEND2 tend to recur and may require multiple re-resections. Like ependymoma their defining histologic feature is perivascular pseudorosettes and a tendency for discrete borders with uninvolved brain tissue and only local tumor cell invasion if any. The latter likely contributes to their relatively indolent biological behavior. Indeed, many cases of EET MN1-BEND2 and other NET MN1 methylation class tumors were originally diagnosed as ependymoma or anaplastic ependymoma [25, 45, 46, 66]. ZFTA fusion-positive supratentorial ependymomas with alternate (non-*RELA*) fusion partners form satellite subclusters of the RELA Ependymoma methylation class and include tumors demonstrating astroblastoma-like histologic features, further supporting that the latter are within the spectrum of ependymal differentiation [26].

Many genes involved in fusions or mutated in the ependymal astroblastoma-like tumors discussed in this review are chromatin remodelers and/or transcriptional regulators affecting DNA methylation and gene expression. This suggests perturbations effecting DNA methylation and downstream chromatin and transcriptional regulation are important factors in pediatric CNS tumorigenesis, perhaps particularly ependymomagenesis. DNA damage causing double strand breaks repaired by error prone non-homologous and alternative end joining [76], particularly involving chromosomes X, 11, and 22, may lead to gene translocations in ependymomagenesis.

Fusions between chromosome 22.12 to 22.13 genes in NET-PATZ1 may be generated by a type of genomic instability called chromothripsis: a process in which catastrophic chromosomal instability leads to clustered deletions and rearrangements within a particular chromosome. Chromothripsis may also be responsible for generating chromosome 22q fusions in rare supratentorial astroblastoma-like tumors lacking *MN1* alterations [67] and chromosome 11q13.1 gene fusions in *ZFTA-RELA* harboring supratentorial ependymomas [26]. Chromosome X chromothripsis may possibly facilitate *MN1-BEND2* fusion in some cases of EET MN1-BEND2 [12, 65]. Characteristic gene fusions in such tumors may drive their oncogenic phenotypes. Chromothripsis itself may be initiated by mutations in SWI/SNF chromatin remodeling proteins or mismatch repair proteins [77].

Mutations in histone modifying proteins may also be important in pediatric ependymomagenesis. DNA methylation and histone deacetylation are intimately linked. In addition to methyl-CpG binding proteins, *DNA methyltransferase 1* (DNMT1), which maintains genomic DNA methylation, also recruits histone deacetylase [78]. SWI/SNF remodeling proteins recognize acetylated or methylated histones and alter nucleosome structure to allow transcription [79]. In astrocytomas, a hypermethylated genomic DNA state (CpG island methylator phenotype or CIMP) in isocitrate dehydrogenase (*IDH1/2*) mutant tumors correlates with increased histone methylation, altered gene expression and improved patient survival [80, 81]. Mutant IDH1/2 causes elevated levels of 2-hydroxyglutarate, which inhibits histone demethylases and the TET family of 5-methlycytosine hydroxylases leading to increased histone and DNA methylation, respectively [82]. *IDH*-mutant tumors, thus, have a better prognosis than *IDH1/2* wildtype astrocytomas.

Inappropriate hypomethylation of growth factor genes such as *IGF2* and other imprinted genes may be an important factor in driving EET MN1-BEND2 tumorigenesis [10]. Altered gene promoter methylation could possibly be secondary to chromatin regulatory gene mutation resulting in chromatin structural changes that effect the activity of DNA methyltransferases [10, 83, 84]. Thus, chromatin structural regulation including by DNA methylation and post-translational modifications of histone proteins may be particularly important in pediatric CNS tumorigenesis.

## Supplementary Information

Below is the link to the electronic supplementary material.

Supplementary file1 (PDF 204 KB)

## Footnotes

1 Not to be confused with DNA methylation.

2 Because the N-terminal methionine is cleaved from histone proteins, H3K28 has historically been referred to as H3K27, with clinical usage of H3K28 occurring only recently [3].

3 Updated data.

4 EET MN1-BEND2 may show folding of central vessels within pseudorosettes, which is not to be confused with true microvascular proliferation [12, 17]

## Declarations

### Data availability

All data is contained within the article and cited references.
