# Supplementary material for: Early ependymal tumor with MN1-BEND2 fusion: a mostly cerebral tumor of female children with a good prognosis that is distinct from classical astroblastoma
Source: J Neurooncol. 2023 Jan 6;161(3):425–39. doi: 10.1007/s11060-022-04222-1 (PMC9992034; doi:10.1007/s11060-022-04222-1)
Supplement: Supplementary file 1 — Supplementary file1 (PDF 204 KB) [file 11060_2022_4222_MOESM1_ESM.pdf]

Table S1: Summary of *MN1*-altered and related tumors in literature

Case #	Sex	Age	Tumor location	Methylation Diagnosis	MN1 break apart FISH (or EWSR1 FISH)	Sequencing	Reference
***MN1:BEND2* fusion**							
18	F	5	Frontal lobe	N/A	N/A	MN1:BEND2 fusion (RNA-seq)	Lake et al., 2020 [1]
dkfz_ABM_15-0004	F	N/A	N/A	CNS_NET-MN1	N/A	*MN1:BEND2* fusion (RNA-seq)	Sturm et al., 2016 [2]
dkfz_CNS-PNET_15-0280	F	8	Occipital lobe	CNS_NET-MN1	*MN1*-rearranged	*MN1:BEND2* fusion (RNA-seq)	
dkfz_EPN_15-0032	F	15	Supratentorial (NOS)	CNS_NET-MN1	*MN1*-rearranged	*MN1:BEND2* fusion (RNA-seq)	
13	F	15	Parietooccipital	CNS_NET-MN1	N/A	*MN1:BEND2* fusion (RNA-seq + RT-PCR + qPCR)	Boisseau et al., 2019 [3]
1	F	6	L parietal; multifocal recurrences	N/A	*MN1:BEND2* fusion by dual-fusion probe	*MN1:BEND2* fusion (RNA-seq + RT-PCR)	Burford et al., 2018 [4]
1	F	4	R frontal lobe	CNS_NET-MN1	N/A	*MN1:BEND2* fusion (DNA-seq)	Jamshidi et al., 2022 [5]
1	F	9	L frontoparietal	N/A	N/A	*MN1:BEND2* fusion	Chen et al., 2020 [6]
C3	F	16	L parietooccipital	CNS_NET-MN1	*MN1*-rearranged	*MN1:BEND2* fusion (RNA-seq)	Lehman et al., 2022 [7]
C10	F	12	R frontoparietal	CNS_NET-MN1	*MN1*-rearranged	*MN1:BEND2* fusion (RNA-seq)	
C13	F	9	R frontoparietal	CNS_NET-MN1	*MN1*-rearranged	*MN1:BEND2* fusion (RNA-seq)	
C21	F	4	L parietooccipital	CNS_NET-MN1	*MN1*-rearranged	*MN1:BEND2* fusion (RNA-seq)	
C29	F	18	L parietal lobe	CNS_NET-MN1	*MN1*-rearranged	*MN1:BEND2* fusion (RNA-seq)	
C44	F	9	R parietal lobe	CNS_NET-MN1	N/A	*MN1:BEND2* fusion (RNA-seq)*	
1†	F	9.3	L frontoparietal	CNS_NET-MN1	N/A	*MN1:BEND2* fusion (RT-PCR)	Lubieniecki et al. 2022 [8]
2	F	10.8	L frontoparietal	N/A	N/A	*MN1:BEND2* fusion (RT-PCR)	
3†	F	6.1	L parietal lobe	CNS_NET-MN1	N/A	*MN1:BEND2* fusion (RT-PCR)	
4†	F	4.9	R parietal lobe	N/A	N/A	*MN1:BEND2* fusion (RT-PCR)	
6	F	12	Spinal cord (T3 to L5)	N/A	N/A	*MN1:BEND2* fusion (RT-PCR)	

***EWSR1-BEND2* fusion and/or *EWSRI* rearrangement by FISH (fusion status not reported)**							
1	M	3M	Brainstem/spinal cord (medulla to C4)	CNS_NET-MN1	*EWSR1* and *BEND2* colocalization detected	*EWSR1-BEND2* fusion (RNA-seq + RT-PCR)	Yamasaki et al., 2020 [9]
1	M	36	Spinal cord (T3 to T5)	CNS_NET-MN1	*EWSR1* and *BEND2* colocalization detected	N/A	Tsutsui et al., 2020 [10]
1	M	20	Brainstem (NOS)	*EWSR1-BEND2 (subclass)*	N/A	*EWSR1-BEND2* (DNA-seq)	Lucas et al., 2021 [11]
2	F	6	Cervical spinal cord	*EWSR1-BEND2 (subclass)*	N/A	*EWSR1-BEND2* (DNA-seq)	
3	F	26	R frontal lobe	*EWSR1-BEND2 (subclass)*	N/A	*EWSR1-BEND2* (DNA-seq)	
4	F	6	L frontal lobe	*EWSR1-BEND2 (subclass)*	N/A	*EWSR1-BEND2* (DNA-seq)	
1	N/A	N/A	Spinal cord (NOS)	N/A	N/A	*EWSR1-BEND2* (DNA-seq)	Ramkissoon et al., 2017 [12]
1	M	38	Brainstem (Cervicomedullary)	CNS_NET-MN1	N/A	*EWSR1-BEND2* (RNA-seq)	Smith-Cohn et al., 2021 [13]
5†	F	6.1	Spinal cord (C3 to T8)	CNS_NET-MN1	*EWSR1*-rearranged	N/A	Lubieniecki et al. 2022 [8]
***MN1:CXXC5* fusion**							
dkfz_CNS-PNET_15-0184	M	16	Parietal lobe	CNS_NET-MN1	*MN1*-rearranged	*MN1:CXXC5* fusion (RNA-seq)	Sturm et al., 2016 [2]
19	M	3	Temporal lobe	N/A	N/A	*MN1:CXXC5* fusion (RNA-seq)	Lake et al., 2020 [1]
1	F	36	L parietal lobe	N/A	N/A	*MN1:CXXC5* fusion (DNA-seq)	Wallace et al., 2022 [14]
**HGNET-MN1 methylation class and/or MN1 rearrangement by FISH (fusion status not reported)**							
dkfz_ABM_15-0001	F	14	Frontotemporal	CNS_NET-MN1	N/A	N/A	Sturm et al., 2016 [2]
dkfz_ABM_15-0002	F	40	L frontal lobe	CNS_NET-MN1	N/A	N/A	
dkfz_ABM_15-0003	F	N/A	N/A	CNS_NET-MN1	N/A	N/A	
dkfz_ABM_15-0005	F	N/A	N/A	CNS_NET-MN1	N/A	N/A	
dkfz_ABM_15-0006	F	7	R parietal lobe	CNS_NET-MN1	N/A	N/A	
dkfz_ABM_15-0008	F	27	L parietal lobe	CNS_NET-MN1	N/A	N/A	
dkfz_ABM_15-0009	F	16	R frontal lobe	CNS_NET-MN1	N/A	N/A	
dkfz_ABM_15-0010	F	19	Frontal lobe	CNS_NET-MN1	N/A	N/A	
dkfz_ABM_15-0011	F	10	Temporal lobe	CNS_NET-MN1	*MN1*-rearranged	N/A	
dkfz_ABM_15-0012	F	3	Frontal lobe	CNS_NET-MN1	*MN1*-rearranged	N/A	
dkfz_ABM_15-0013	F	25	N/A	CNS_NET-MN1	N/A	N/A	

dkfz_ABM_15-0014	F	18	Intraventricular	CNS_NET-MN1	N/A	N/A	
dkfz_ABM_15-0015	F	7	R central region	CNS_NET-MN1	N/A	N/A	
dkfz_ABM_15-0016	F	19	Parietal lobe	CNS_NET-MN1	*MN1*-rearranged	N/A	
dkfz_ABM_15-0017	F	17	Parietooccipital	CNS_NET-MN1	*MN1*-rearranged	N/A	
dkfz_CNS-NET_15-0001	F	6	R hemispheric (NOS)	CNS_NET-MN1	N/A	N/A	
dkfz_CNS-PNET_15-0130	F	N/A	N/A	CNS_NET-MN1	*MN1*-rearranged	N/A	
dkfz_CNS-PNET_15-0144	F	2	R parietal lobe	CNS_NET-MN1	*MN1*-rearranged	N/A	
dkfz_CNS-PNET_15-0200	F	20	R frontal lobe	CNS_NET-MN1	N/A	N/A	
dkfz_CNS-PNET_15-0235	F	14	L frontotemporal	CNS_NET-MN1	N/A	N/A	
dkfz_CNS-PNET_15-0274	F	8	N/A	CNS_NET-MN1	*MN1*-rearranged	N/A	
dkfz_CNS-PNET_15-0297	F	N/A	N/A	CNS_NET-MN1	N/A	N/A	
dkfz_CNS-tumor_15-0001	F	11	L frontal lobe	CNS_NET-MN1	*MN1*-rearranged	N/A	
dkfz_EPN_15-0020	F	5	Frontal lobe	CNS_NET-MN1	N/A	N/A	
dkfz_EPN_15-0021	F	5	Frontal lobe	CNS_NET-MN1	N/A	N/A	
dkfz_EPN_15-0024	F	22	Frontobasal	CNS_NET-MN1	N/A	N/A	
dkfz_EPN_15-0031	F	17	Spinal (C2)	CNS_NET-MN1	N/A	N/A	
dkfz_ETMR_15-0013	F	N/A	Hemispheric (NOS)	CNS_NET-MN1	N/A	N/A	
dkfz_GBM_15-0009	F	13	Thalamic	CNS_NET-MN1	Negative	N/A	
dkfz_MNG_15-0012	F	36	R temporooccipital	CNS_NET-MN1	N/A	N/A	
dkfz_CNS-PNET_15-0131	M	11	Frontal lobe	CNS_NET-MN1	*MN1*-rearranged	N/A	
dkfz_CNS-PNET_15-0229	M	11	Parieto-central	CNS_NET-MN1	N/A	N/A	
dkfz_EPN_15-0018	M	11	Cerebellar	CNS_NET-MN1	N/A	N/A	
dkfz_EPN_15-0028	M	N/A	Posterior fossa	CNS_NET-MN1	N/A	N/A	
dkfz_EPN_15-0035	M	31	Temporal lobe	CNS_NET-MN1	N/A	N/A	
dkfz_CNS-PNET_15-0106	N/A	N/A	N/A	CNS_NET-MN1	N/A	N/A	
dkfz_EPN_15-0026	N/A	N/A	N/A	CNS_NET-MN1	N/A	N/A	

1	F	16	Frontoparietal	N/A	*MN1*-rearranged	N/A	Mhatre et al., 2019 [15]
2	F	23	Parietal lobe	N/A	*MN1*-rearranged	N/A	
4	F	14	Parietal lobe	N/A	*MN1*-rearranged	N/A	
5	F	27	Frontal lobe	N/A	*MN1*-rearranged	N/A	
6	F	10	Parietal lobe	N/A	*MN1*-rearranged	N/A	
1	F	6	L frontal lobe	N/A	*MN1*-rearranged	N/A	Hirose et al., 2018 [16]
2	F	6	Occipital lobe	N/A	*MN1*-rearranged	N/A	
3	F	18	R frontal lobe	N/A	*MN1*-rearranged	N/A	
4	F	24	L frontal lobe	N/A	*MN1*-rearranged	N/A	
8	F	37	L occipital lobe	N/A	*MN1*-rearranged	N/A	
1	F	4	L parietal lobe	CNS_NET-MN1	N/A	N/A	Petruzzellis et al., 2019 [17]
1	F	6	L parietal lobe	N/A	*MN1*-rearranged	N/A	Fudaba et al., 2020 [18]
1	F	36	Brainstem (Cervicomedullary)	CNS_NET-MN1	N/A	N/A	Gopakumar et al., 2022 [19]
1	7	F	L parietal lobe	CNS_NET-MN1	*MN1*-rearranged	N/A	Sari et al., 2021 [20]
1	M	11	Dorsal brainstem (pontomedullary)	N/A	*MN1*-rearranged	N/A	Shin et al., 2018 [21]
1	M	35	L frontal lobe	Unclassifiable	*MN1*-rearranged	CDKN2A/B deep deletion,<br>TERT promoter mutation (DNA-seq)	Wood et al., 2018 [22]
3	F	9	R frontal lobe	CNS_NET-MN1	*MN1*-rearranged	NA	
4	F	10	L parietal lobe	CNS_NET-MN1	*MN1*-rearranged	NA	
8	F	31	L parietal lobe	CNS_NET-MN1	*MN1*-rearranged	ATM mutation, NF2 structural rearrangement (DNA-seq)	
1	F	20	Spinal cord, T1-T2	N/A	*MN1*-rearranged	N/A	Yamada et al., 2018 [23]
28	N/A	7	V3	CNS_NET-MN1	Four cases were *MN1*rearrang-ed; one case was uninterpretable	N/A	Pages et al., 2019 [24]
29	N/A	4	Frontal lobe	CNS_NET-MN1		N/A	
30	N/A	11	Frontal lobe	CNS_NET-MN1		N/A	
31	N/A	6	Temporoparietal	CNS_NET-MN1		N/A	
32	N/A	17	N/A	CNS_NET-MN1		N/A	

42	M	36	Spinal cord, thoracic	CNS_NET-MN1	Negative	N/A	Neumann et al., 2020 [25]
43	F	11	Supratentorial (NOS)	CNS_NET-MN1	Rearranged	N/A	
C32	F	12	Cerebral (NOS)	CNS_NET-MN1	Rearranged	N/A	Lehman et al., 2019 [26]
C33	F	13	Parietal lobe	CNS_NET-MN1	Rearranged	N/A	
C34	F	3	Parietal lobe	CNS_NET-MN1	Rearranged	N/A	
1	F	4.8	R parietal lobe	CNS_NET-MN1	N/A	N/A	Baroni et al., 2020 [27]
2	F	5.8	L parietal lobe	CNS_NET-MN1	N/A	N/A	
3	F	8.9	L parietal lobe	CNS_NET-MN1	N/A	N/A	
4	F	5	Cervicothoracic spinal	CNS_NET-MN1	N/A	N/A	
5	F	4.5	R frontal lobe	CNS_NET-MN1	N/A	N/A	
6	F	7	L parietooccipital	CNS_NET-MN1	N/A	N/A	
7	F	3.6	L parietooccipital	CNS_NET-MN1	N/A	N/A	
8	F	6.7	R parietal lobe	CNS_NET-MN1	N/A	N/A	
9	M	14.6	Cervicothoracic spinal	CNS_NET-MN1	N/A	N/A	
10	F	13	R frontoparietal	CNS_NET-MN1	N/A	N/A	
11	M	36	Spinal cord	CNS_NET-MN1	N/A	N/A	
12	F	10	L frontoparietal	CNS_NET-MN1	N/A	N/A	
13	F	8	L parietal lobe	CNS_NET-MN1	N/A	N/A	
14	F	14	R parietal lobe	CNS_NET-MN1	N/A	N/A	
C7	F	33	L temporal lobe	CNS_NET-MN1	Rearranged	N/A	Lehman et al. 2022 [7]
C43	F	10	R parietal lobe	CNS_NET-MN1	N/A	N/A	

*RNA sequencing was performed as part of the clinical testing (unpublished).
†These cases were also included in the series by Baroni et al. [27] listed above.
*RNA sequencing was performed as part of the clinical testing (unpublished).
Abbreviations: N/A, not performed or reported; NOS, not otherwise specified.

References

1. Lake JA, Donson AM, Prince E, Davies KD, Nellan A, Green AL, Mulcahy Levy J, Dorris K, Vibhakar R, Hankinson TC, Foreman NK, Ewalt MD, Kleinschmidt-DeMasters BK, Hoffman LM, Gilani A (2020) Targeted fusion analysis can aid in the classification and treatment of pediatric glioma, ependymoma, and glioneuronal tumors. Pediatr Blood Cancer 67:e28028. doi:10.1002/pbc.28028
2. Sturm D, Orr BA, Toprak UH, Hovestadt V, Jones DTW, Capper D, Sill M, Buchhalter I, Northcott PA, Leis I, Ryzhova M, Koelsche C, Pfaff E, Allen SJ, Balasubramanian G, Worst BC, Pajtler KW, Brabetz S, Johann PD, Sahm F, Reimand J, Mackay A, Carvalho DM, Remke M, Phillips JJ, Perry A, Cowdrey C, Drissi R, Fouladi M, Giangaspero F, Lastowska M, Grajkowska W, Scheurlen W, Pietsch T, Hagel C, Gojo J, Lotsch D, Berger W, Slavc I, Haberler C, Jouvet A, Holm S, Hofer S, Prinz M, Keohane C, Fried I, Mawrin C, Scheie D, Mobley BC, Schniederjan MJ, Santi M, Buccoliero AM, Dahiya S, Kramm CM, von Bueren AO, von Hoff K, Rutkowski S, Herold-Mende C, Fruhwald MC, Milde T, Hasselblatt M, Wesseling P, Rossler J, Schuller U, Ebinger M, Schittenhelm J, Frank S, Grobholz R, Vajtai I, Hans V, Schneppenheim R, Zitterbart K, Collins VP, Aronica E, Varlet P, Puget S, Dufour C, Grill J, Figarella-Branger D, Wolter M, Schuhmann MU, Shalaby T, Grotzer M, van Meter T, Monoranu CM, Felsberg J, Reifenberger G, Snuderl M, Forrester LA, Koster J, Versteeg R, Volckmann R, van Sluis P, Wolf S, Mikkelsen T, Gajjar A, Aldape K, Moore AS, Taylor MD, Jones C, Jabado N, Karajannis MA, Eils R, Schlesner M, Lichter P, von Deimling A, Pfister SM, Ellison DW, Korshunov A, Kool M (2016) New brain tumor entities emerge from molecular classification of CNS-PNETs. Cell 164:1060-1072. doi:10.1016/j.cell.2016.01.015
3. Boisseau W, Euskirchen P, Mokhtari K, Dehais C, Touat M, Hoang-Xuan K, Sanson M, Capelle L, Nouet A, Karachi C, Bielle F, Guegan J, Marie Y, Martin-Duverneuil N, Taillandier L, Rousseau A, Delattre JY, Idbaih A (2019) Molecular profiling reclassifies adult astroblastoma into known and clinically distinct tumor entities with frequent mitogen-activated protein kinase pathway alterations. Oncologist 24:1584-1592. doi:10.1634/theoncologist.2019-0223
4. Burford A, Mackay A, Popov S, Vinci M, Carvalho D, Clarke M, Izquierdo E, Avery A, Jacques TS, Ingram WJ, Moore AS, Frawley K, Hassall TE, Robertson T, Jones C (2018) The ten-year evolutionary trajectory of a highly recurrent paediatric high grade neuroepithelial tumour with MN1:BEND2 fusion. Sci Rep 8:1032. doi:10.1038/s41598-018-19389-9
5. Jamshidi P, McCord M, Horbinski C, Jennings L, Santana dos Santos L, Fudyma IA, DeCuypere M, Yap KL, Rathbun P, Wadhwani N (2022) Methylation profiling improves the care of pediatric brain tumor patients. AJSP: Reviews & Reports 27:e1-e4. doi:10.1097/pcr.0000000000000493
6. Chen W, Soon YY, Pratiseyo PD, Sutanto R, Hendriansyah L, Kuick CH, Chang KTE, Tan CL (2020) Central nervous system neuroepithelial tumors with MN1-alteration: an individual patient data meta-analysis of 73 cases. Brain Tumor Pathol 37:145-153. doi:10.1007/s10014-020-00372-0
7. Lehman NL, Spassky N, Sak M, Webb A, Zumbar CT, Usubalieva A, Alkhateeb KJ, McElroy JP, Maclean KH, Fadda P, Liu T, Gangalapudi V, Carver J, Abdullaev Z, Timmers C, Parker JR, Pierson CR, Mobley BC, Gokden M, Hattab EM, Parrett T, Cooke RX, Lehman TD, Costinean S, Parwani A, Williams BJ, Jensen RL, Aldape K, Mistry AM

(2022) Astroblastomas exhibit radial glia stem cell lineages and differential expression of imprinted and X-inactivation escape genes. Nat Commun 13:2083. doi:10.1038/s41467-022-29302-8
8. Lubieniecki F, Vazquez V, Lamas GS, Camarero S, Nuñez FJ, Baroni L, Schüller U, Alderete D (2022) The spectrum of morphological findings in pediatric central nervous system MN1-fusion-positive neuroepithelial tumors. Childs Nerv Syst. doi: 10.1007/s00381-022-05741-y
9. Yamasaki K, Nakano Y, Nobusawa S, Okuhiro Y, Fukushima H, Inoue T, Murakami C, Hirato J, Kunihiro N, Matsusaka Y, Honda-Kitahara M, Ozawa T, Shiraishi K, Kohno T, Ichimura K, Hara J (2020) Spinal cord astroblastoma with an EWSR1-BEND2 fusion classified as a high-grade neuroepithelial tumour with MN1 alteration. Neuropathol Appl Neurobiol 46:190-193. doi:10.1111/nan.12593
10. Tsutsui T, Arakawa Y, Makino Y, Kataoka H, Minamiguti S, Hirose T, Nobusawa S, Nakano Y, Ichimura K, Miyamoto S (2020) PATH-23. Adult spinal cord astroblastoma with EWSR1-BEND2 fusion. Neuro-Oncology 22:iii429-iii429. doi:10.1093/neuonc/noaa222.658
11. Lucas CG, Gupta R, Wu J, Shah K, Ravindranathan A, Barreto J, Gener M, Ginn KF, Prall OWJ, Xu H, Kee D, Ko HS, Yaqoob N, Zia N, Florez A, Cha S, Perry A, Clarke JL, Chang SM, Berger MS, Solomon DA (2022) EWSR1-BEND2 fusion defines an epigenetically distinct subtype of astroblastoma. Acta Neuropathol 143:109-113. doi:10.1007/s00401-021-02388-y
12. Ramkissoon SH, Bandopadhayay P, Hwang J, Ramkissoon LA, Greenwald NF, Schumacher SE, O'Rourke R, Pinches N, Ho P, Malkin H, Sinai C, Filbin M, Plant A, Bi WL, Chang MS, Yang E, Wright KD, Manley PE, Ducar M, Alexandrescu S, Lidov H, Delalle I, Goumnerova LC, Church AJ, Janeway KA, Harris MH, MacConaill LE, Folkerth RD, Lindeman NI, Stiles CD, Kieran MW, Ligon AH, Santagata S, Dubuc AM, Chi SN, Beroukhim R, Ligon KL (2017) Clinical targeted exome-based sequencing in combination with genome-wide copy number profiling: precision medicine analysis of 203 pediatric brain tumors. Neuro Oncol 19:986-996. doi:10.1093/neuonc/now294
13. Smith-Cohn M, Abdullaev Z, Aldape K, Quezado M, Rosenblum M, Vanderbilt C, Rodriguez F, Laterra J, Eberhart C (2021) Molecular clarification of brainstem astroblastoma with EWSR1-BEND2 fusion in a 38-year-old man. Free Neuropathol 2. doi:10.17879/freeneuropathology-2021-3334
14. Wallace GC, Macaulay RJB, Etame AB, Aldape K, Pina Y (2022) Histopathologically atypical astroblastoma with MN1-CXXC5 fusion transcript diagnosed by methylation classifier. Arch Community Med Public Health 8:113-117. doi:10.17352/2455-5479.000185
15. Mhatre R, Sugur HS, Nandeesh BN, Chickabasaviah Y, Saini J, Santosh V (2019) MN1 rearrangement in astroblastoma: study of eight cases and review of literature. Brain Tumor Pathol 36:112-120. doi:10.1007/s10014-019-00346-x
16. Hirose T, Nobusawa S, Sugiyama K, Amatya VJ, Fujimoto N, Sasaki A, Mikami Y, Kakita A, Tanaka S, Yokoo H (2018) Astroblastoma: a distinct tumor entity characterized by alterations of the X chromosome and MN1 rearrangement. Brain Pathol 28:684-694. doi:10.1111/bpa.12565

17. Petruzzellis G, Alessi I, Colafati GS, Diomedi-Camassei F, Ciolfi A, Pedace L, Cacchione A, Carai A, Tartaglia M, Mastronuzzi A, Miele E (2019) Role of DNA methylation profile in diagnosing astroblastoma: A case report and literature review. Front Genet 10:391. doi:10.3389/fgene.2019.00391
18. Fudaba H, Momii Y, Kawasaki Y, Goto H, Nobusawa S, Fujiki M (2020) Well-differentiated astroblastoma with both focal anaplastic features and a meningioma 1 gene alteration. NMC Case Rep J 7:205-210. doi:10.2176/nmccrj.cr.2020-0028
19. Gopakumar S, McDonald MF, Sharma H, Tatsui CE, Fuller GN, Rao G (2022) Recurrent HGNET-MN1 altered (astroblastoma MN1-altered) of the foramen magnum: Case report and molecular classification. Surg Neurol Int 13:139. doi:10.25259/SNI_1208_2021
20. Sari R, Altinoz MA, Ozyar E, Danyeli AE, Elmaci I (2021) A pediatric cerebral tumor with MN1 alteration and pathological features mimicking carcinoma metastasis: may the terminology "high grade neuroepithelial tumor with MN1 alteration" still be relevant? Childs Nerv Syst 37:2967-2974. doi:10.1007/s00381-021-05289-3
21. Shin SA, Ahn B, Kim SK, Kang HJ, Nobusawa S, Komori T, Park SH (2018) Brainstem astroblastoma with MN1 translocation. Neuropathology 38:631-637. doi:10.1111/neup.12514
22. Wood MD, Tihan T, Perry A, Chacko G, Turner C, Pu C, Payne C, Yu A, Bannykh SI, Solomon DA (2018) Multimodal molecular analysis of astroblastoma enables reclassification of most cases into more specific molecular entities. Brain Pathol 28:192-202. doi:10.1111/bpa.12561
23. Yamada SM, Tomita Y, Shibui S, Takahashi M, Kawamoto M, Nobusawa S, Hirato J (2018) Primary spinal cord astroblastoma: case report. J Neurosurg Spine 28:642-646. doi:10.3171/2017.9.SPINE161302
24. Pages M, Pajtler KW, Puget S, Castel D, Boddaert N, Tauziede-Espariat A, Picot S, Debily MA, Kool M, Capper D, Sainte-Rose C, Chretien F, Pfister SM, Pietsch T, Grill J, Varlet P, Andreiuolo F (2019) Diagnostics of pediatric supratentorial RELA ependymomas: integration of information from histopathology, genetics, DNA methylation and imaging. Brain Pathol 29:325-335. doi:10.1111/bpa.12664
25. Neumann JE, Spohn M, Obrecht D, Mynarek M, Thomas C, Hasselblatt M, Dorostkar MM, Wefers AK, Frank S, Monoranu CM, Koch A, Witt H, Kool M, Pajtler KW, Rutkowski S, Glatzel M, Schuller U (2020) Molecular characterization of histopathological ependymoma variants. Acta Neuropathol 139:305-318. doi:10.1007/s00401-019-02090-0
26. Lehman NL, Usubalieva A, Lin T, Allen SJ, Tran QT, Mobley BC, McLendon RE, Schniederjan MJ, Georgescu MM, Couce M, Dulai MS, Raisanen JM, Al Abbadi M, Palmer CA, Hattab EM, Orr BA (2019) Genomic analysis demonstrates that histologically-defined astroblastomas are molecularly heterogeneous and that tumors with MN1 rearrangement exhibit the most favorable prognosis. Acta Neuropathol Commun 7:42. doi:10.1186/s40478-019-0689-3
27. Baroni LV, Rugilo C, Lubieniecki F, Sampor C, Freytes C, Nobre L, Hansford JR, Malalasekera VS, Zapotocky M, Dodgshun A, Martinez OC, La Madrid AM, Lavarino C, Sunol M, Rutkowski S, Schuller U, Bouffet E, Ramaswamy V, Alderete D (2020) Treatment response of CNS high-grade neuroepithelial tumors with MN1 alteration. Pediatr Blood Cancer 67:e28627. doi:10.1002/pbc.28627
